# Factors Affecting Soil Fauna Feeding Activity in a Fragmented Lowland Temperate Deciduous Woodland

**DOI:** 10.1371/journal.pone.0029616

**Published:** 2012-01-03

**Authors:** Jake E. Simpson, Eleanor Slade, Terhi Riutta, Michele E. Taylor

**Affiliations:** 1 Centre for Ecology and Hydrology, Natural Environment Research Council, Wallingford, Oxfordshire, United Kingdom; 2 Department of Biology, Imperial College London, London, United Kingdom; 3 Wildlife Conservation Research Unit, Department of Zoology, University of Oxford, Oxford, Oxfordshire, United Kingdom; 4 Environmental Change Institute, School of Geography and the Environment, University of Oxford, Oxford, Oxfordshire, United Kingdom; University of Utah, United States of America

## Abstract

British temperate broadleaf woodlands have been widely fragmented since the advent of modern agriculture and development. As a result, a higher proportion of woodland area is now subject to edge effects which can alter the efficiency of ecosystem functions. These areas are particularly sensitive to drought. Decomposition of detritus and nutrient cycling are driven by soil microbe and fauna coactivity. The bait lamina assay was used to assess soil fauna trophic activity in the upper soil horizons at five sites in Wytham Woods, Oxfordshire: two edge, two intermediate and one core site. Faunal trophic activity was highest in the core of the woodland, and lowest at the edge, which was correlated with a decreasing soil moisture gradient. The efficiency of the assay was tested using four different bait flavours: standardised, ash (*Fraxinus excelsior* L.), oak (*Quercus robur* L.), and sycamore (*Acer pseudoplatanus* L.). The standardised bait proved the most efficient flavour in terms of feeding activity. This study suggests that decomposition and nutrient cycling may be compromised in many of the UK's small, fragmented woodlands in the event of drought or climate change.

## Introduction

Leaf litter decomposition is a key step in the cycling of carbon and nutrients, and is achieved by the activity of soil microbes and fauna working in tandem. Decomposition by soil microbes is facilitated by the mechanical and metabolic degradation of litter by soil invertebrates [1]–[4]. Trophic activity of soil fauna is constrained by physical and biological factors: soil temperature and moisture [5], soil profile structure [6], the presence of above ground leaf litter [7], soil chemistry (following fertilisation, liming or pollution) [8], [9], substrate quality [10], [11] and soil fauna community composition [12]. Soil moisture and temperature are particularly variable with proximity to woodland edges [13], and therefore may influence the feeding activity of soil invertebrates.

Since the advent of modern agriculture and industrial and urban development, British forests have been extensively fragmented. They now cover approximately 12% of the UK land area compared to 75% of continuous forest 6000 years ago, with 75% of wooded areas being less than 2 ha and consisting of largely edge habitat [14], [15]. The forest microclimate at woodland edges is drier and warmer than forest interior, this is brought about by both the direct and indirect effects (i.e. greater rate of transpiration) of increased solar radiation and air turbulence [13], [16], [17]. This microclimate is known to affect some invertebrate communities in woodland areas up to 1 km from the edge [18] therefore it should not be assumed that ecosystem functions (e.g. soil turnover and nutrient cycling) are uniform across the whole woodland.

Soil fauna feeding activity can be used as an indicator of the biological status of soil [7]. Given the complexity of soil fauna ecology whereby the same group can function at different trophic levels, general patterns in community composition which influence decomposition in a predictable way are yet to be discovered [1]. For simplicity, a general index of biological activity can be measured using soil fauna feeding activity alone (rather than attempting to measure decomposition by all the component organisms in the system). To measure soil fauna activity as a function of decomposition, the bait lamina assay was developed by Von Törne [19]. This technique has been used in microcosm [12], mesocosm [3] and *in situ* investigations [7], with great success in ecotoxicological studies [8], [20]. The first bait lamina studies acknowledged that it was difficult to disentangle the effects of fauna and micro-organisms on feeding activity. However, recent studies both in microcosms and in the field have shown that macrofauna, such as earthworms [20], mesofauna, such as enchytraeids, and microarthropods, such as collembola [12] and acari [7] are the main feeders on bait lamina. Specifically, these studies have concluded that while some microbial activity does occur, it is small compared with the faunal activity [12]. Moreover, Gongalsky et al. [6] suggest that micro-organisms cannot contribute to perforation during the short durations typically used in bait lamina studies.

Gestel et al. [3] found that while alternative methods, such as litter bags or cotton sticks, are mainly indicative of the activity of soil microbes the bait lamina method is the most direct measure of the activity of the soil fauna. Moreover, with bait lamina sticks faunal feeding activity can be measured across different depth profiles.

Soil fauna have demonstrated preferential feeding for a nettle-based substrate in laboratory experiments [12], whereas no significant preference for different grass-based bait was found *in situ* in arid grassland in Canada [10]. Leaf litter from the dominant tree species can be incorporated into bait to examine the *in situ* relationship between local dominant tree species and faunal feeding preference [21]. Lumbricid worms have been shown to prefer leaf litter from *Fraxinus* species to *Tilia* species, and their abundance can be correlated with the palatability of the leaf litter [22]. However, it is unknown whether baits made from local tree species are more efficient indicators of trophic activity than the standardised-bait in temperate broadleaf woodland ecosystems.

To the best of our knowledge, this study is the first to experimentally investigate the effect of changes in abiotic conditions at the forest edge on the feeding activity of soil invertebrates using the bait lamina method. It is hypothesised that feeding activity will differ significantly at the woodland edges compared to the core, where soil moisture and temperature differ correspondingly. This study is also the first to compare standardised and bait made from local tree leaf litter in the bait lamina assay. Bait is hypothesised to be preferentially fed upon if it is made of the local dominant tree leaf litter compared to the standardised-bait. Furthermore, trophic activity is expected to be greatest towards the soil surface, where food is most abundant for detritivores.

## Materials and Methods

### Site description

The experiment was conducted at Wytham Woods (51°46′N, 001°20′W, UK National Grid SP 461 081), Oxfordshire. The woodland is approximately 400 ha and is characterised by ancient and secondary woodland. The woodland is a ‘mixed deciduous’ woodland community W8 according to the National Vegetation Classification (*Fraxinus excelsior* L.- *Acer campestre* L. - *Mercurialis perennis* L. woodland) [23], [24]. Meteorological records for the site from 1992 to 2009 show the mean annual temperature to be 10.1°C, and an average precipitation of 730 mm (Environmental Change Network data, obtained from Centre for Ecology and Hydrology, Wallingford, UK, 2010), which is typical of the Central England climate.

### Experimental design

Bait lamina sticks (Terra Protecta GmbH, Berlin, Germany) are thin PVC strips (1 mm×6 mm×120 mm) with sixteen apertures (1.5 mm diameter) bored at 5 mm intervals [21]. The apertures are filled with either standardised bait or bespoke bait, whereby bran flakes are substituted with local dried and ground leaf material. The strips are placed vertically into the soil, and feeding activity is assessed by comparing the number of apertures that have been perforated versus the number left untouched during the exposure period [21]. The bait lamina sticks are sold with a standardised-bait containing cellulose powder, bran flakes and activated carbon (70∶27∶3). Ash (*Fraxinus excelsior*), oak (*Quercus robur*), and sycamore (*Acer pseudoplatanus*) leaves were collected in the autumn of 2008 using raised nets in the woods, and were ground using a ball grinder into a fine-grade powder. Three bait flavours were prepared using the leaf powder, cellulose powder and activated carbon (to the same ratio as the standardised bait with ground leaves replacing the bran flakes). Distilled water was added to the bait powder to form a thick paste. The apertures on the sticks were filled manually (by compressing the paste between the finger and thumb, then running the stick through it) and then allowed to dry at room temperature. A second application was required because the bait contracts on drying. Each aperture was verified as being filled completely by holding the sticks against a light source after the bait had dried following the second application. In total 360 sticks were used (90 of each flavour).

Five sites were selected: the southern edge (SE), 100 m from the southern edge towards the core (S100m), within the woodland core (Core), the northern edge (NE), and 100 m from the northern edge (N100m). Sites were considered to be spatially independent; the distance between the core and the northern and southern sites was 0.9 km and 1.4 km respectively, and between the northern and southern edges 2.1 km. Edge sites were located within 5 m of the perimeter fence of the woods. As soil type has a significant effect on soil fauna activity [5], each site is on the same Denchworth series clay soil, a surface water gley soil in the England and Wales soil Survey Classification [25]. Results from a 18 ha forest monitoring plot (within which the core plot is sited) indicated there is little variability in physical properties or chemical analysis across the Denchworth soil series [26]. At each of the edge and 100 m edge sites, three replicate plots (40 cm×40 cm) were marked approximately15 m apart along a transect parallel to the woodland edge. In the core site [27] the three plots were marked randomly within a 1 ha area, approximately 25 m apart. The leaf litter was left undisturbed, leaving the natural litter depth at each plot. Each plot was divided into a grid of four columns and three rows, forming 12 cells, with two replicate sticks in each. Each column contained one of four bait flavour treatments (standard, sycamore, ash or oak), and the rows signified the exposure time of the bait lamina sticks (9, 19 or 34 days), although time data were pooled together for analysis. To insert the sticks into the soil without dislodging the bait in the apertures, or breaking the sticks, a modified metal knife created a slit 10 cm deep, with the same width and thickness as the bait lamina sticks. The shallowest aperture was at 1.2 cm, and the deepest at 8.7 cm. The sticks were installed on 14^th^ October 2009. To test whether soil invertebrates are the agents responsible for making the perforations in the sticks a control test was conducted in the woods. A container was filled with defaunated soil (freeze treated to kill invertebrates), and 36 sticks (9 per flavour) were placed in the soil and exposed for the same periods as the experimental sticks. There were no perforations of any of the apertures of these sticks after any of the time intervals verifying the method.

Environmental data were collected throughout the exposure periods. On installation of the bait lamina sticks, soil moisture and temperature were measured with a hand held 12 cm TDR probe (HydroSense, Campbell Scientific Ltd., Shepshed, U.K.) and temperature probe at five locations around the plot to give a mean reading for each plot (15 readings per site). The soil temperature and moisture were not measured within the plot to avoid disturbing the sampling area. Soil moisture and temperature readings were taken on seven subsequent occasions during the exposure period. Continuous data for air temperature, rainfall and soil temperature (10 cm) for Wytham were obtained from an automatic weather station (AWS, Didcot Instruments Co., Abingdon, U.K.) at a nearby grassland site. Soil pH was measured after the exposure period by taking ten 10 cm deep soil samples from each site, mixing them together and taking three separate pH readings on the field moist samples, on 1: 2.5 extracts in water [28] to achieve a mean pH for the site.

### Statistical Analysis

The environmental conditions were analysed across the five sites for soil temperature, moisture, and pH separately using general linear models. A Spearman's rank test was used (because the data were non-parametric) to test if soil moisture and temperature were correlated.

Feeding activity was analysed as the proportion of holes perforated and the data was arcsine square-root transformed. To account for temporal pseudoreplication (the same sites were revisited at different intervals) the average number of holes perforated per flavour per plot over the three separate time periods was taken. Using generalized linear additive models, location and bait flavour were modelled as fixed effects with plot within location as a random effect and soil moisture as a covariate. Soil temperature was excluded as a covariate as because no significant difference was observed between sites. Depth was included in the model with a cubic regression spline smoother due to the nonlinear relationship between feeding activity and depth. To account for the heterogeneity of variances among locations and bait flavours a separate variance structure for each location and flavour was applied. Alternative models were compared using likelihood ratio tests and AIC values (a model with simple variance structure vs. different variances per strata; a model with one general smoother for depth vs. smoothers by individual locations vs. smoothers by groups of locations). The optimal model with different variances for location and flavour and three smoothers for depth (Core; N100m and S100m; NE and SE) was selected. Analyses were carried out using R(GUI) [29] nlme and mcgv packages.

## Results

### Environmental Conditions

**Figure 1:**
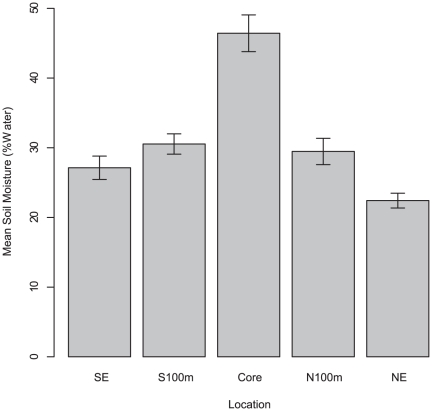
Mean soil moisture across sites (±SE). The bars represent the soil moisture data taken on 8 occasions during 34 day sampling period.

Over the 34 day sampling period mean soil temperature did not vary significantly among the sites (F_5, 121_ = 1.55, P = 0.18). The average soil moisture was significantly higher at the core than the 100 m and edge plots (F_5, 121_ = 21.86, P<0.001, figure 1). Soil moisture and temperature were negatively correlated (ρ = −0.58, P<0.001). Mean soil pH (6.85±0.51) was anti-logged and did not differ significantly across sites (F_4, 5_ = 2.54, P = 0.061).

The air temperature at Wytham for the preceding month and during the sampling period was similar to the long term average. Rainfall though was particularly low in September 2009 (9.0 mm compared to 65.4 mm, 1992–2008), but average for October and November combined.

### Factors affecting soil fauna feeding activity

Feeding activity varied between 0–75% (perforations per stick), with an overall mean feeding activity of 8.5% for all flavours and all sites. Moisture, location and bait flavour and depth were the principal factors influencing feeding activity (table 1). Trophic activity was lowest at the edges and highest in the core.

**Table 1:** Optimal generalized additive mixed model: feeding as a function of moisture, location, bait flavour and depth.

Explanatory variable	Degrees of freedom	F value	P value
Moisture	1	8.132	<0.001
Location	4	2.229	0.064
Flavour	3	10.598	<0.001
Location*Flavour	12	14.562	<0.001
Smoother Core	3.720	9.339	<0.001
Smoother N100m and S100m	1.004	40.468	<0.001
Smoother NE and SE	3.968	12.752	<0.001

Depth was modelled with cubic regression spline smoothers. Note that the degrees of freedom and P values for the smoothers are estimates. The higher the degrees of freedom the more non-linear the smoother (df = 1 indicates a linear smoother).

**Figure 2:**
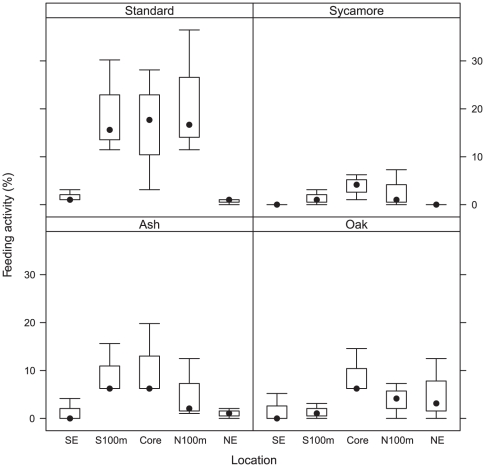
Feeding activity for each bait flavour across the five sampling sites in Wytham Woods. The black dots represent the median, with the boxes representing the 25^th^ and 75^th^ percentiles. The range is given by the whiskers.

Feeding preferences were different among the sites. Overall, standard bait was preferred, especially in the core and 100 m sites, while edges showed more variability (figure 2, table 1). The standard bait represented 9% of holes perforated at the northern edge, compared to its highest proportion of 62% at the N100m site.

**Figure 3:**
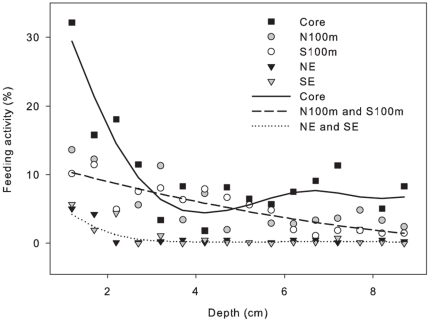
Feeding activity at different depths below the soil surface. The proportion is total number of holes perforated at each depth, averaged across bait flavours and plots within each site. The smoothers are derived from the generalised additive mixed model (see Statistical analysis). The model validation showed that the N100m and S100m sites could be described with one smoother as could the SE and NE sites.

Feeding activity tended to be highest near the surface, with an average of 18% and 14% holes perforated at 1.2 cm and 1.7 cm respectively (figure 3). The locations could be divided into three groups based on the feeding activity pattern: Feeding activity had the steepest depth gradient in the core, intermediate at the 100 m sites and the least pronounced at the edge sites.

## Discussion

### Edge effects on feeding activity

The results demonstrate that feeding activity decreases towards the edge of the woodland. Soil moisture was a significant term in the minimal adequate model. Feeding activity was strongly correlated with the decreasing moisture gradient which occurs with proximity to the edge [16]. Gongalsky et al. [5] found soil temperature to have a greater influence on feeding than soil moisture, but when soil temperature does not significantly vary between sites, soil moisture has been shown to be the principle abiotic driver of trophic activity.

As feeding activity at 100 m was similar to the core, it is suggested that the feeding is not significantly affected by woodland edge effects at this distance. Chen et al. [30] found soil moisture variability was highest within 15 m from the edge. Most of the feeding activity occurred within 2 cm of the soil surface, as found by similar studies using bait lamina sticks [7], [8], [10], and feeding at the edges was found to be especially concentrated at the surface, compared with at the core where higher levels of feeding occurred at all depths. This may be due to lower soil moisture at the edges which causes the clay soil to harden, in turn limiting the vertical movement of soil invertebrates. Eggleton et al. [31] demonstrated that a reduction in soil moisture has a significant negative effect on the abundance of epigeic (surface feeding) and endogeic (sub-terrestrial feeding) species.

The edge effect is not limited to the direct abiotic factors considered in this study, but also includes a variety of indirect biotic factors which are beyond the scope of this study [32]. Using our model, the spatial difference in soil fauna feeding activity can be attributed to the moisture component of the edge effect. The results have demonstrated that feeding at the edge is significantly lower at the edges than sites 100 m towards the woodland interior and beyond. As soil fauna play a key role in nutrient cycling and decomposition processes [4], [11] this has important applications to small wooded areas which have a high edge to core ratio. Therefore, these processes may be significantly reduced as soil fauna feeding activity decreases towards the drier woodland edges.

### Bait flavours and feeding activity

The standard bait proved to be the best indicator of trophic activity compared to the bespoke baits (mean feeding activity of 16% and 6% respectively). Field studies using the standard bait alone have tended to report overall higher trophic activity than those using bespoke baits. An average of 3% activity over 65 days was observed in Canadian soils using only bespoke baits [10], however it is difficult to draw comparisons between previous studies due to abiotic and biotic differences in sampling sites. For the purposes of assessing spatial heterogeneity in feeding activity efficiently and comparably, it is suggested that only the standardised bait is used.

### Effect of the environmental conditions

Overall feeding activity was lower than expected according to the bait lamina manufacturers [33], but Von Törne [19] observed comparable feeding activity of 4–16% with standard bait in German soils. The month leading up to the exposure period was comparatively dry, causing the clay soil to harden and crack. These conditions are not conducive to invertebrate activity, especially for earthworms that favour moist soil and thus, and could have caused the overall low feeding activity [31]. The relatively dry period preceding the study may have extenuated the moisture limiting condition at the woodland edge, but these short drought periods are not out of the ordinary and climate models predict drier summers and/or increased frequency of summer droughts in the UK [34], [35].

Feeding activity is known to occur down to temperatures of 5°C [5] which is lower than the minimum observed soil temperature in this experiment (8.5°C). While soil temperature was demonstrated to have an important effect on feeding activity in laboratory studies [5], the mean soil temperature between sites did not differ significantly, and therefore its effects could not be attributed to the variance observed in this study.

The soil at the surface, if not insulated by leaf litter, is very prone to desiccation which in turn may have pronounced effects in reducing nutrient turnover as a result of decreased trophic activity [4], [7], [11]. While small visual differences in the depth of leaf litter were noted between the sites, leaf litter manipulation studies have demonstrated that additional layers (i.e. increasing the depth of leaf litter) have little effect on soil temperature and moisture compared to natural litter depths [36]. Therefore, any differences in leaf litter depth between the sites are unlikely to have affected feeding activity. Soil acidity is known to affect the distribution of both earthworms and soil arthropods [9], [37], [38], but analysis of soil pH across the five sampling sites and the control show no significant difference, and therefore it is assumed that soil pH did not contribute to differences in feeding activity.

This study emphasises the possible effects of climate change on soil fauna feeding activity with reduced soil moisture, particularly in small, fragmented woodlands. Consistent with our results, studies conducted using the litter bag method have shown that low precipitation and moisture deficit reduces litter decomposition rates [39]–[42].Litter decomposition is a key process in ecosystem carbon cycle and is estimated to contribute up to 70% to the annual carbon efflux [43], [44]. Given our results, future estimates of ecosystem functions (i.e. litter decomposition by soil fauna) and services (i.e. climate regulation) of temperate forests in a fragmented landscape should account for heterogeneity in soil fauna feeding activity as a result of the edge effect.
